# Cheerios Effect Inspired Microbubbles as Suspended and Adhered Oral Delivery Systems

**DOI:** 10.1002/advs.202004184

**Published:** 2021-02-24

**Authors:** Cheng Zhao, Lijun Cai, Min Nie, Luoran Shang, Yongan Wang, Yuanjin Zhao

**Affiliations:** ^1^ Department of Rheumatology and Immunology Institute of Translational Medicine The Affiliated Drum Tower Hospital of Nanjing University Medical School Nanjing 210002 China; ^2^ State Key Laboratory of Bioelectronics School of Biological Science and Medical Engineering Southeast University Nanjing 210096 China; ^3^ Zhongshan‐Xuhui Hospital The Shanghai Key Laboratory of Medical Epigenetics the International Co‐laboratory of Medical Epigenetics and Metabolism Ministry of Science and Technology, and Institutes of Biomedical Sciences Fudan University Shanghai 200032 China; ^4^ State Key Laboratory of Toxicology and Medical Countermeasures Institute of Pharmacology and Toxicology Academy of Military Medical Sciences Beijing 100850 China

**Keywords:** Cheerios effect, drug delivery, microbubble, microfluidics, systemic lupus erythematosus

## Abstract

Oral drug administration has an important role in medical treatment. Attempts to develop drug microcarriers with desired features for extended duration and improved absorption is highly sought. Herein, inspired by the physical phenomenon of the Cheerios effect, a novel microfluidic electrospray microbubble carrier is presented that can suspend and actively adhere to the stomach for durable oral delivery. Compared with conventional fabrication methods, the present strategy shows stability and controllability of the product. Benefiting from their uniform hollow structure, the resultant microbubbles present the same behavior of the Cheerios and can float in the gastric juice, adhere and remain to the stomach wall, which thus enhance the duration and absorption of the loaded drugs. Based on these, it is demonstrated as a proof of concept that the dexamethasone‐loaded hollow microbubbles can be applied to oral administration and remain suspended and adhered to the stomach of murine for more than 1 d, showing good therapeutic effect in treating lupus erythematosus. Thus, it is believed that the microbubbles floating system will find important values in long‐term oral administration.

## Graphical abstract

The cheerios effect inspired microbubbles are presented through microfluidic electrospray with features of suspending and actively adhering to stomach. These microbubbles can stay in the stomach without relying on water and can also stick to the stomach after the liquid is empty. Thus, it contributes to improved values in long‐term oral administration.

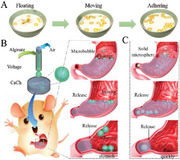

## 1 Introduction

Long‐term administration is one of the most used therapies in the treatment of chronic diseases, such as systemic lupus erythematosus (SLE), hypertension, etc.^[^ ^1^, ^2^, ^3^, ^4^ ^]^ Insights regarding how to simplify the administration methods and enhance the bioavailability of drugs have been gained to reduce the frequency of administration, and thus relieving chronic disease patients from tedious operation and mental pressure.^[^ ^5^, ^6^, ^7^, ^8^, ^9^, ^10^ ^]^ To reach these goals, a host of approaches have been developed, such as invasive treatment, oral administration, transdermal administration, etc.^[^ ^11^, ^12^, ^13^, ^14^, ^15^ ^]^ Among them, oral administration is strongly preferred because it does not require a professional skill that allows patients to self‐administer drugs conveniently without dependence on the doctor.^[^ ^16^, ^17^, ^18^, ^19^ ^]^ Especially, by encapsulating drugs into different kinds of carrier materials, the specificity, controllable release profile, and localized effect of the drug could be effectively improved.^[^ ^20^, ^21^, ^22^, ^23^ ^]^ Although with many successes, the duration and absorption of the drug in an oral administration process are significantly affected by the motility of the gastrointestinal and digestive system, both of which result in low drug dissolution, quick emptying, and irregular absorption, thus greatly limiting the efficacy of oral administration for long‐term use.^[^ ^16^, ^24^, ^25^ ^]^ Therefore, a novel strategy to prolong the duration time of the carriers and enhance their drug effect in oral administration for long‐term use is highly anticipated.

**Figure 1:**
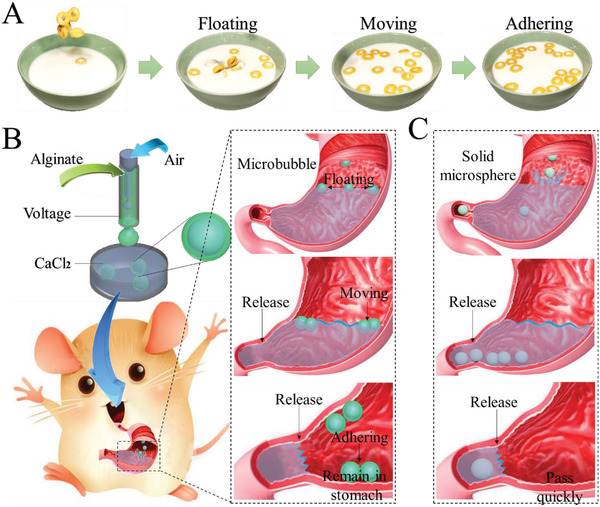
Design and fabrication of the Cheerios effect inspired microbubbles. A) The schematic diagram of the Cheerios effect phenomenon. Cheerios float on the surface of the fluid and adhere to the wall. B) The microbubbles were fabricated through electrostatic microfluidics. After gavage, the microbubbles could adhere to the stomach wall and remain in the stomach after stomach emptying. C) The conventional solid microspheres sank to the bottom of the stomach and flowed into the intestine during stomach emptying.

In this paper, inspired by the phenomenon of the “Cheerios effect,” we presented a liquid‐independent floating drug microcarrier based on hollow microbubbles for long‐term oral delivery, as shown in **Figure** **1** Generally, the cheerios could float on the surface of a liquid as a result of buoyancy. Especially, when the wall of the liquid container is hydrophilic, the cheerios can actively move toward the highest point of the liquid under the jointed effects of buoyancy and capillarity (Figure S1, Supporting Information). This provides inspiration for the design of a suspended drug carrier system, where we envisioned microbubbles to show the same Cheerios effect in the gastrointestinal tract. Microbubbles, which possess low density, show the same properties as cheerios in a fluid environment, serving as an ideal carrier for designing novel floating drug delivery system.^[^ ^26^, ^27^ ^]^ However, a key bottleneck in applying microbubbles as drug carriers is their fabrication. Although microfluidic techniques, among others, have shown great promise in fabricating microemulsions or microparticles based on their precise manipulation of microscale fluids, the generation of microbubbles is difficult because of gas compression in the microfluidic microchannel. In addition, the floating gas would readily destroy the stability between the multiphase fluids in the microfluidics.^[^ ^28^, ^29^, ^30^, ^31^, ^32^ ^]^ Therefore, new approaches to fabricating microbubbles with high stability and controllability as drug microcarriers are still desirable.

Herein, we used electrostatically driven microfluidics to generate drug‐loaded microbubbles showing the Cheerios effect by floating and adhering to the stomach as a novel oral delivery system. By combining a two‐phase flow (a liquid sheath flow and a gaseous core flow) microfluidics with an electrospray setup, microbubbles were generated and encapsulated within an alginate hydrogel shell through instant ionic crosslinking under mild conditions without organic solvent. Notably, the electrostatic drive reduced the dependence on the liquid shear force and reduced the compression of the gas. Besides, the rapid gelation of the shell largely improved the stability of the microbubbles. Moreover, the electrostatically driven microfluidic platform enabled precise control of the structure of microbubbles by thorough design of the fluid phases.^[^ ^33^, ^34^, ^35^, ^36^ ^]^ It was demonstrated that benefiting from the hollow structure, the resultant microbubbles presented the same behavior of cheerios and could suspend in and adhere to the wall of stomach, thus greatly increasing their retention time. Compared with solid carriers and other commercially available bubble materials, drug loaded in the microbubble shell layer would not be emptied and thus showed a prolonged dissolution and release in the gastric fluid, which could increase the drug absorption efficiency. As a proof of concept, the dexamethasone‐loaded hollow microbubbles floating system was used for oral administration in an SLE murine model. It was found that the presented system could suspend and adhere to the stomach of murine for more than 1 d and thus showing a good therapeutic effect in treating SLE. These results indicated that the microbubbles floating system has infinite potential in long‐term oral administration.

## 2 Results and Discussion

**Figure 2:**
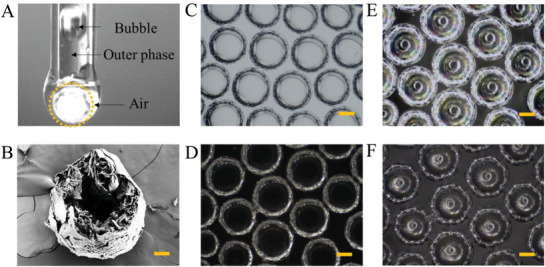
Fabrication and characterization of the microbubbles. A) The real‐time image of the microfluidic electrospray process for fabricating microbubble. B) SEM image of half dissected microbubble. Scale bar is 100 µm. C–F) The light source irradiates the microbubbles at different distances to produce different emitted light. Scale bars are 100 µm. C) The light source was located 1 cm directly below the bottom of the microbubble. D) The ring light source illuminates the microbubbles from the same horizontal plane. E) The light source was located 5 cm above the microbubble. F) The light source was located 10 cm above the microbubble. Scale bars are 100 µm in (C)–(F).

In a typical experiment, the hollow microbubbles were fabricated in a coaxial capillary microfluidic chip with the combination of electrospray (**Figure** **2**A). As alginate has a large number of carboxyl groups and exhibits superhydrophilic properties (Figure S2, Supporting Information), it was chosen as the shell material so that the microbubbles could simulate the Cheerios effect. To be specific, an air flow was set as the inner phase and an aqueous solution of alginate mixed with polyvinyl alcohol (PVA) was used as the outer phase. In the microfluidic chip, the inner air phase was sheathed by the alginate phase and formed a co‐flow regime. Then, droplets containing air bubbles were generated and collected in 2% CaCl_2_ gelling bath under an electrostatic field. Fast gelation between Ca^2+^ and alginate led to immediate encapsulation of air bubbles and formed a core–shell structure, as shown in Figure 2B,C. The features of the microbubble were further characterized by a scanning electron microscope (SEM). The results showed that the cut microbubbles possessed a ring‐shaped shell and a hollow core, indicating a favorable hollow structure. The smooth surface of the microbubbles resembles that of a mirror and exhibited strong light reflection. Therefore, the microbubbles, which were transparent, displayed a hexagonal appearance by reflecting the wall of the dish inside (Figure 2C). Moreover, at different illumination angles, the microbubbles showed different color reflections from solid microspheres owing to their unique optical properties, which all proved the hollow construction of microbubbles (Figure 2C–F). Furthermore, the size of the microbubbles could be precisely controlled by regulating the voltage and flowing volume. It was noted that when the voltage was reduced, the product gradually changed from single‐core to multicore microbubbles. In addition, the size of the internal microbubbles could be controlled by adjusting the flow rate ratio of the external phase to the internal phase. Briefly, a larger ratio leads to a smaller size (Figure S3, Supporting Information).

**Figure 3:**
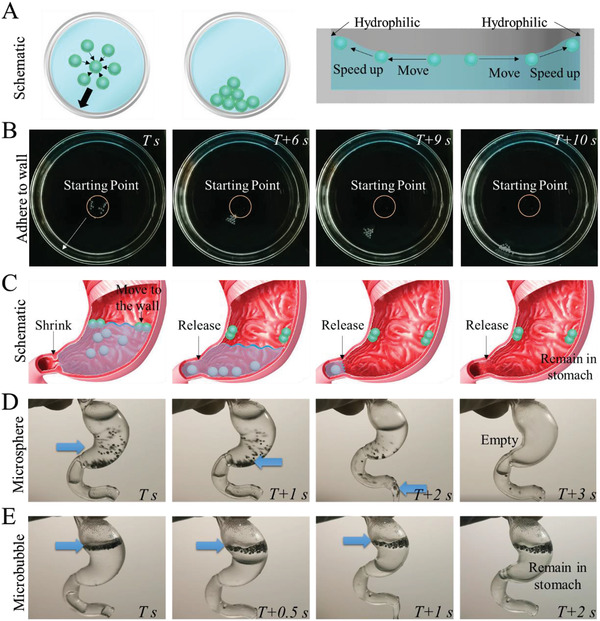
Characterization of microbubbles’ Cheerios effect for gastric retention. A) The mechanism of Cheerios effect on floating microbubbles in liquid. B) Real‐time images of the microbubbles in liquid. C,D) The microspheres and microbubbles were analyzed in actual anatomy using a 3D‐printed stomach model. C) Schematic illustration of the process of microbubbles and microspheres in the stomach. D) Simulated process of conventional solid microspheres in the 3D‐printed stomach model. E) Simulated process of microbubbles in 3D‐printed stomach model. A total of 40 microspheres and microbubbles were used in (D) and (E).

To identify the Cheerios effect of microbubbles, the as‐prepared microbubbles were settled in a glass dish and a simulated pylorus. To simulate the actual situation, we analyzed the surface properties of the stomach wall through contact angle measurement. Owing to the folds on the surface of the stomach wall, it looked uneven, which was not like the smooth surface of a glass slide. However, the hydrophilicity of the glass dish makes it suitable for in vitro simulation as the surface of the stomach wall was superhydrophilic (Figure S2B,C, Supporting Information). In this experiment, the fabricated microbubbles were able to quickly reunite on the liquid surface through buoyancy and then adhere to the wall of the dish in a short time (**Figure** **3**A,B and Video S1, Supporting Information). On the contrary, solid microspheres sank at the bottom of the dish and kept this state for 15 min (Figure S4A,B, Supporting Information). To further analyze the impact of the size of the microbubbles on their floating behavior, a 3 mm microbubble and a 300 µm microbubble were prepared and settled in the dish. In this experiment, the larger microbubble moved faster outward and adhered to the wall of the dish (Figure S5, Supporting Information). However, small microbubbles could aggregate and move collectively at a higher speed (Figure 3B and Figure S5B, Supporting Information). Therefore, considering the need for in vivo experiments, we selected smaller microbubbles for further experiments.

To better simulate the behavior of the microbubbles in the actual anatomy, a 3D‐printed stomach model was built to analyze the intragastric process of conventional solid microspheres and microbubbles (Figure 3C–E). During this process, the conventional solid microspheres sank quickly in the stomach, leading them to be emptied by the liquid (Figure 3D). This may be the reason why conventional solid microspheres are difficult to stay in the stomach. In the experimental group, however, the microbubbles were capable of floating on the liquid surface as a result of buoyancy, and reuniting and actively sticking to the stomach wall owing to the Cheerios Effect, which increased the friction between the microbubbles and the stomach wall. When the liquid was emptied, the microbubbles were retained on the surface of the stomach wall owing to increased friction. Thus, even though the liquid was emptied, the microbubbles could still stay in the stomach, indicating that the microbubbles could retain in the stomach in a liquid‐independent manner (Figure 3E). Moreover, when the liquid passed through the simulated pylorus, the microbubbles could still remain on the wall while solid microspheres quickly slipped away from the simulated pylorus without hindrance (Figure S4C–E and Video S2, Supporting Information).

Although many studies have used microbubbles for drug delivery applications, few of them have been engaged in the digestive system owing to the dilemma of easy degradation and a short retention time. In addition, conventional bubble fabrication methods lack a good control of the bubble stability, size distribution, and, most importantly, the resultant bubbles did not show the special flow dynamics as the microbubbles proposed in this paper. For example, SonoVue is a well‐known microbubble formulation that has been used in clinic and clinical researches.^[^ ^37^ ^]^ To explore the difference between the clinically used microbubbles and the Cheerios effect inspired microbubbles, we performed an analysis of the flow dynamics and stomach adhesion ability on SonoVue. We observed that SonoVue were mostly suspended rather than floated owing to the high density of sulfur hexafluoride inside (Figure S6A, Supporting Information). The stomach adhesion ability was further characterized in the 3D‐printed model, and the microbubbles in SonoVue passed the stomach quickly (Figure S6B, Supporting Information). In addition to the inappropriate density, the small size of the SonoVue (2.5 µm) microbubbles also impeded their motion ability and thus made them incapable of showing the Cheerios effect. In addition, SonoVue was very difficult to carry drugs owing to its lipid membrane. So we did not conduct further evaluation of its therapeutic efficacy. Overall, an optimized drug carrier for long‐term oral administration requires well‐design and precise control of the material, size, and density of the microbubbles, which poses high requirements for the preparation process. Therefore, the present microbubbles, with their size, density, and fluid dynamics finely controlled by microfluidic electrospray, were suitable for long‐term drug delivery in the digestive tract.

**Figure 4:**
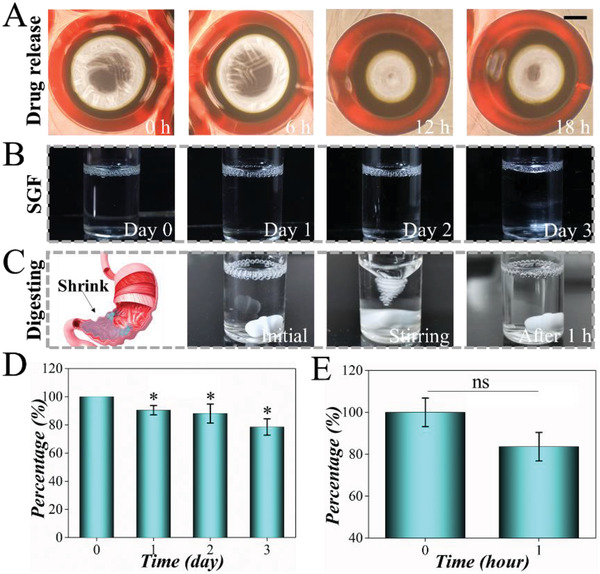
Analysis of the stability and drug release ability of microbubbles in SGF. A) Microbubbles with 4% PVA were characterized under a confocal microscope. Scale bar is 100 µm. B) Images of microbubbles cocultured in SGF for 3 d. C) Culturing microbubbles in SGF and stirring with a stirrer to simulate gastric digestion for 1 h. D) The quantification of numbers of microbubbles in the static state. E) The quantification of numbers of microbubbles during digesting (*n* = 20). *t*‐test was used for comparison with the 0 d (ns, nonsignificant; *p < 0.05).

To investigate the drug release ability and stability of microbubbles in practical conditions, the microbubbles were cultured in simulated gastric fluid (SGF) for analysis. As surfactants may affect the pharmaceutical process, we analyzed the effect of PVA concentration on the drug release abilities of microbubbles. Microbubbles with different concentrations of PVA were fabricated, and their entrapment efficiency and 90‐h drug release profiles were tested (Figure S7A,B, Supporting Information). They showed no significant difference in entrapment efficiency. Moreover, they all showed relatively slow drug release. Microbubbles with 4% PVA were chosen for further research as they exhibited a quick response in the first 24 h and a sustained release later. We then recorded the drug release process through confocal microscopy. Results showed that microbubbles with 4% PVA maintained good shape and controlled release in SGF for 18 h (**Figure** **4**A). To analyze the stability of the microbubbles in gastric fluid, we cultured the microbubbles in SGF for 3 d. During this process, the microbubbles were able to float stably on the surface of SGF (Figure 4B,C). In addition, we simulated the stability of the microbubbles in the SGF during peristalsis. Under high‐speed stirring, the microbubbles could keep an intact structure and float stably on the surface (Figure 4D,E). Owing to the reaction between alginate and gastric fluid, the microbubbles could exist stably and remain intact in the form of an alginic microbubble in the stomach as reported in our previous work.^[^ ^38^ ^]^ However, owing to the dissolution and release of gas in water, the microbubbles gradually became smaller. In the simulated digestion process, the microbubbles gradually changed from spherical to elliptical owing to vigorous stirring (Figure S8, Supporting Information). These results suggested that the microbubbles could carry out stable drug release in SGF, which made them an ideal carrier for gastric retention and oral administration.

**Figure 5:**
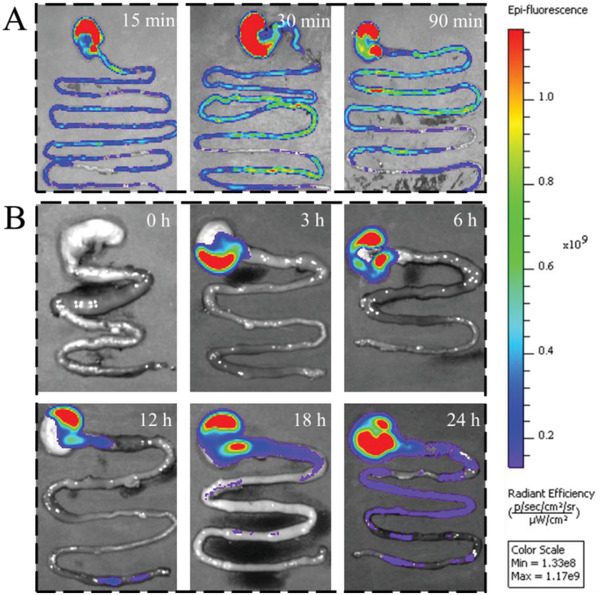
IVIS of the digestive tract after administration of microspheres and microbubbles. A) The rats’ intestines of the microsphere control group were taken out for imaging at 15, 30, and 90 min after administration. B) The rats’ intestines of the microbubble group were taken out for imaging at 0, 3, 6, 12, 18, and 24 h after administration.

In practical situations, the conditions in the digestive tract are relatively changeable, which leads to unpredictable behavior of the microcarriers. Therefore, the distribution of microbubbles in the body was also analyzed by using rats as an example. In this experiment, the solid microsphere group was set as a control. After administration of the microspheres and microbubbles, the rats’ stomachs and intestines were taken out at the set time. Then, the samples were imaged by an in vivo imaging system (IVIS) to analyze the distribution of the carriers in the digestive tract. According to our assumptions, the microsphere group passed the stomach faster, and the microbubble group passed the stomach slower. Therefore, different observation time points were designed for different groups. In the control group, the microspheres were quickly excreted into the intestine. In addition, owing to the function of the pylorus, the whole process was much slower than that in the in vitro model. After 15 min, a part of the microspheres has been discharged into the intestine. After 30 min, most microspheres have entered the intestine. At 90 min, most of the microspheres in the stomach have been eliminated, many of which have entered the intestine (**Figure** **5**A). In the microbubble group, the microbubbles remained in the stomach and did not enter the intestine in the first 6 h. With the peristalsis of the stomach, the microbubbles began to enter the intestine after 12 h, and the portion of the microbubbles entering the intestine began to increase after 24 h. At the beginning, the fluorescence was different owing to the different stomach morphology. Compared with the microsphere group, the microbubble group showed a significant retention effect, suggesting the potential of microbubbles in providing a longer‐lasting sustained release capability. Before in vivo experiments, mesenchymal stem cells (MSCs) were used to assess the cytotoxicity of the microbubbles (Figure S9, Supporting Information). After 3 d of culture, cells in the microbubble groups proliferated well and so did the control group, which indicated the great biocompatibility of the microbubbles and microspheres. Based on these results, these microbubbles were envisioned to be ideal carriers for drug loading and delivery.

**Figure 6:**
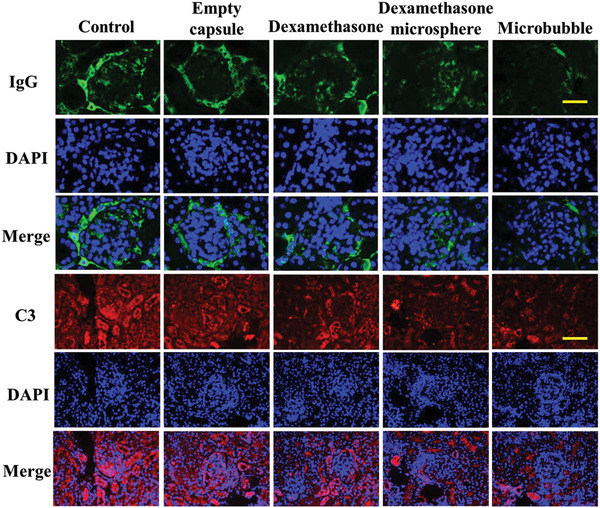
Effect of microbubbles on MRL/*lpr* mice. Mice were treated with PBS, empty capsules, dexamethasone, dexamethasone microsphere, and dexamethasone microbubbles. Representative images of kidney IgG and C3 immunostaining in glomerulus. Scale bars are 50 µm in C3 and 30 µm in IgG.

To investigate the potential value of the microbubbles, an SLE murine model was constructed with MRL/*lpr* mice which spontaneously develop severe nephritis. The mice were divided into five groups and administrated with phosphate buffered saline (PBS), empty microbubble, dexamethasone, dexamethasone microsphere, and dexamethasone microbubble. From the microscale observation of immune staining, the hematoxylin and eosin (H&E) slides of the control group showed that the glomerular shape disappeared, and the kidney was completely infiltrated by inflammatory cells which had darker staining. The results of the empty capsule group were consistent with the PBS group. The condition of the dexamethasone group was slightly better, and the inflammatory infiltration was relatively weak, while the dexamethasone microsphere group could slightly see the structure of the glomerulus. In the microbubble group, the infiltration of inflammatory cells was significantly reduced, and the glomerular shape was relatively clear (Figure S10A, Supporting Information). This indicates the efficacy of the microbubbles in the treatment of SLE, alleviating inflammation, and treating renal dysfunction associated with SLE. The main manifestation of nephritis is the deposition of antibodies and complement in the glomeruli, and the expression of IgG and C3 in the kidney indicates the degree of immune stress. To further verify the therapeutic effect of microbubbles on pathological changes in SLE, fluorescence immunoassay was used to analyze the deposition of IgG and C3 in the kidney. In the PBS group and empty capsule group, the level of IgG and C3 is very high, indicating the damage to the kidney. Dexamethasone and dexamethasone microspheres can relieve the performance to a certain extent. In the microbubble group, the glomerulus showed minimal deposition of IgG and C3 (**Figure** **6**). The deposition of IgG and C3 was further quantified. The result showed significant reduction of markers in the kidney through loading drug in the carriers, especially in the microbubble's group. It indicated that the microbubble can better improve the absorption and utilization of drugs in the body (Figure S10B,C, Supporting Information). These results demonstrated that the microbubbles were ideal carriers for treating SLE through extending the drug duration, reducing the inflammation and finally restoring organs’ function.

## 3 Conclusion

In conclusion, we have proposed a Cheerios effect inspired microbubble from microfluidic electrospray for long‐term drug delivery. These microbubbles remained floated and showed collective motion toward the wall of the stomach owing to buoyancy and capillarity. Benefiting from this property, the retention of the microbubbles in the stomach was extended, as confirmed by in vitro experiments. In addition, the drug loading and releasing capacities were optimized. Moreover, the therapeutic effect of the drug‐loaded microbubbles in treating SLE was demonstrated in vivo, with relieved inflammation and reduced deposition of IgG and C3 for preventing organ dysfunction. These features manifested that the microbubbles had great potential in oral administration. It was noted that the acoustic impedance of the microbubbles and body tissues is inconsistent, which makes the microbubbles possible for ultrasound imaging. However, the size of the present microbubbles is significantly larger than that for using as an ultrasound contrast agent. In view of this, father efforts might be directed in tailoring the features of the microbubbles for other biomedical applications.

## 4 Experimental Section

##### Materials and Animals

Alginate, calcium chloride (CaCl_2_), PVA, Rhodamine B, pepsin, phosphate buffer saline, and calcein‐AM (CAM) were bought from Aladdin. Sulfur hexafluoride microbubbles (SonoVue) were bought from Bracco Suisse SA. Fluorescein sodium salt was bought from Macklin. Hydrochloric acid (HCl) and sodium hydroxide (NaOH) were bought from Nanjing Wanqing Chemical Glass Ware and Instrument Co., Ltd. Round capillaries were purchased from Great Wall Scientific Instruments Inc. A high voltage power supply was gained from Dongwen High Voltage Power Supply (Tianjin) Co., Ltd. MRL/*lpr* mice were purchased from the Shanghai SLAC Laboratory Animal Co., Ltd. (Shanghai, P. R. China). SD rats were purchased from the Nanjing Institute of Animal Model.

##### Microfluidic Device Design

To fabricate the microfluidic device, the inner capillary was tapered with a laboratory portable Bunsen burner (Honest MicroTorch) for reaching an inner diameter of ≈100 µm. The inner diameter of the out capillary was 500 µm. Afterward, the capillaries were coaxially assembled, and the connections were fixed with a transparent epoxy resin (Devcon 5 Minute Epoxy).

##### Microfluidic Electrospray Fabrication of Microbubbles

Figure 1B shows the schematic process of microbubbles generated by the microfluidic electrospray system. The inner phase was air, and the outer phase was 1.2% alginate and PVA with different concentrations (0.5, 1, 2, and 4 wt%, respectively). The fluid phases were injected through different syringes and flowed into corresponding inlets of the microfluidic device via polyethylene tubes. During the fabrication process, the flowing rate of the inner phase was 100 µL min^−1^ and the flow rate of the outer phase was 50 µL min^−1^. The flow rates were controlled by a syringe pump (LSP‐01‐2A, LongerPump, China). A 7.5 kV electrostatic potential was applied to generate the microbubbles. The collecting distance was 2 cm. Monodisperse bubbles were formed with the assistance of the shear force of the aqueous phase. Meanwhile, the aqueous phase broke into droplets under an electric field, with microbubbles encapsulated. The droplets with alginate shells were collected in a dish containing 2 wt% CaCl_2_ to allow the quick gelation of alginate. To further analyze the relationship between parameters and microbubbles, the flowing rate and voltage were adjusted. To fabricate microspheres for control, the inner air flow was stopped. The other parameters were the same so that the size and component were the same as that of the microbubbles.

##### Characterization

Bright field images of the microbubbles were recorded by optical microscopy (OLYMPUS IX71) equipped with a CCD camera (DP30BW). The detail of the microstructure of the microbubbles was analyzed through a SEM (HITACHI, S‐3000N).

##### Cheerios Effect In Vitro

The surface properties of the stomach and the glass dish were characterized using a contact angle measuring instrument (GBX D‐I) at 28 °C. Briefly, a 2 µL water drop was placed on the samples, and each sample was tested three times at different sites. Images and angles were recorded. The fabricated microbubbles and microspheres were set in glass dishes with SGF (10 g L^–1^ pepsin and 3.84 mL L^–1^ HCl). Subsequently, the movement process of the microbubbles and microspheres in the dish was recorded. To simulate the process of microcarriers passing through the pylorus, the entire process was simulated and recorded in a pipette in vitro. To investigate the behavior of the microcarriers in actual anatomy, a 3D stomach model was built through Form 3 (Formlabs). Afterward, the microcarriers were placed in the 3D‐printed stomach model, and the emptying processes were recorded.

##### In Vitro Stability and Drug Release

To investigate the stability and drug release ability, the fabricated microbubbles were incubated with SGF. The microbubbles were incubated in static SGF for 3 d to analyze the stability of the microbubbles in the resting state of the stomach. The digestion process of the stomach was simulated through a violent stirring process in which microbubbles were placed to analyze the stability in the digestion process. To analyze the drug release ability, fluorescein sodium was used as a model drug with similar molecular weight and was loaded into the alginate‐PVA pregel solution through direct mixing ahead of the formation of the microbubbles. Subsequently, the microbubbles were collected and cultured in 5 mL SGF at 37 °C. 100 µL fluid was collected for test at 0.5, 1, 1.5, 6, 12, 18, 30, 60, and 90 h. Later, an equal amount of SGF was added for a supplement. The samples were repeated for three replicates, added with an equal amount of NaOH, and tested with a microplate reader at 493 nm. Then, the drug release ability and entrapment efficiency were calculated according to the standard curve. A standard curve was constructed according to the fluorescein isothiocyanate (FITC) of known concentration, and the drug concentration at each time point was obtained by comparing the measured absorbance value with the labeled curve. Hence, the release kinetic process could be evaluated. For further demonstration, 1 mL microbubbles (4% PVA) loaded with rhodamine B were fabricated and incubated in 5 mL SGF at 37 °C. Images were taken with a confocal laser scanning microscope at 0, 6, 12, and 18 h.

##### In Vivo Gastric Retention

IVIS was used to visualize the distribution of microcarriers in the digestive system. Generally, rhodamine B was encapsulated in these microcarriers for imaging. Afterward, experimental SD rats are divided into microsphere group and microbubble group. In the microsphere group, the intestines were collected and imaged with IVIS Spectrum at 15, 30, and 90 min after gavage. In the microbubble group, the intestines were collected and imaged with IVIS Spectrum at 0, 3, 6, 12, 18, and 24 h. All the images were normalized with the image at 0 h which received no microcarriers.

##### Biocompatibility Tests of the Microbubble

The encapsulating materials were added to wells containing 1 mL culture medium and incubated for 24 h. Subsequently, the culture medium was fetched out for cell culture. The MSCs were placed in 96‐well dishes with 4000 cells per well (100 µL) for 72 h. During this process, different 24‐h aged extract solutions were produced for replacement at each time point. Afterward, the cells were stained with CAM to show the living cells on day 1, 2, and 3. Cells were cultured in Dulbecco's modified Eagle medium under the condition of 37 °C and 5% CO_2_.

##### In Vivo Therapeutic Effect

MRL/*lpr* mice were housed under specific pathogen‐free conditions in the animal center of the Affiliated Drum Tower Hospital of Nanjing University Medical School. All animal experiments were approved by the Committee of Experimental Animal Administration of the Affiliated Drum Tower Hospital of Nanjing University Medical School (No. 2019AE01012). Twenty MRL/*lpr* mice were divided into five groups, namely, groups treated with PBS, empty capsule, dexamethasone, dexamethasone microsphere, and dexamethasone microbubble, respectively. The interventions were given at the age of 12 weeks. Dexamethasone was given by 1 mg kg^−1^ three times a week for four weeks. The mice in dexamethasone microsphere and dexamethasone microbubble groups were given the same dose of dexamethasone. After four weeks’ treatment, the mice were sacrificed, and kidneys were collected.

##### H&E and Immunohistochemistry (IHC) Staining

The kidney was collected for H&E staining and IHC staining. First, the collected organs were frozen in liquid nitrogen and treated with optimal cutting temperature embedding matrix (Leica Biosystems, Nussloch, Germany). Subsequently, the sections were cut at a thickness of 7 µm and stained with antibodies, namely, FITC‐anti‐mouse IgG or Fluor594 conjugated goat anti‐rabbit C3 (Santa Cruz Biotechnology, Santa Cruz, CA) followed by the staining of nuclei with 4,6‐diamidino‐2‐phenylindole (DAPI, Sigma). Afterward, diaminobenzidine tetrahydrochloride and Mayer's Hematoxylin were applied for immunostaining. The histological structure of the glomerulus and deposition of IgG and C3 was then assessed.

##### Statistical Analysis

All the data showed were normalized with the control group. The presented data sets were exhibited as mean ± SD. The sample size for each statistical analysis was recorded in the Experimental Section or figure legend. Student's *t*‐test was used for comparison between two groups, and the difference was regarded as statistically significant if *p* < 0.05. All statistical analyses were conducted using SPSS software.

## Conflict of Interest

The authors declare no conflict of interest.

## Supporting information

Supporting Information

Supplemental Movie 1

Supplemental Movie 2

Supplemental Movie 3

## Data Availability

Research data are not shared.
