# Supplementary material for: Cheerios Effect Inspired Microbubbles as Suspended and Adhered Oral Delivery Systems
Source: Adv Sci (Weinh). 2021 Feb 24;8(7):2004184. doi: 10.1002/advs.202004184 (PMC8025035; doi:10.1002/advs.202004184)
Supplement: Supplementary file 1 — Supporting Information [file ADVS-8-2004184-s004.pdf]

WILEY-VCH

© 2021 Wiley-VCH GmbH

ADVANCED SCIENCE
Open Access

Supporting Information

for *Adv. Sci.*, DOI: 10.1002/advs.202004184

Cheerios effect inspired microbubbles as suspended and adhered oral delivery systems

Cheng Zhao, Lijun Cai, Min Nie, Luoran Shang, Yongan Wang*, Yuanjin Zhao**

Supporting Information

Cheerios effect inspired microbubbles as suspended and adhered oral delivery systems

Cheng Zhao, Lijun Cai, Min Nie, Luoran Shang, Yongan Wang*, Yuanjin Zhao**

Author Contributions

Y.J.Z., L.R.S. and Y.A.W. conceived the study and participated in its design. C.Z. conducted the materials' experiment, drew all figures in this article and wrote this manuscript. L.J.C. helped wrote the manuscript. M.N. finished the animal experiment and improved the language of the manuscript.

Fig. S1. Schematic illustration of Cheerios Effect based microbubbles.

Fig. S2. The water contact angle result of the alginate, stomach tissue and glass dish.

Fig. S3. The relationship between the microbubble's morphology and producing parameters.

Fig. S4. The performance of the solid microspheres in the glass dish and the simulated pylorus.

Fig. S5. The performance of microbubbles with different sizes in the dish.

Fig. S6. The flow dynamics and stomach adhesion ability of SonoVue.

Fig. S7. The influence of PVA on entrapment efficiency and drug release.

Fig. S8. The morphology of microbubbles after culturing in static state for 3 days and in digestion for 60 mins.

Fig. S9. Biocompatibility of encapsulating materials.

Fig. S10. Effect of microbubbles on MRL/lpr mice.

Movie S1. Microbubbles actively adhere to the wall based on Cheerios Effect.

Movie S2. The performance of microbubbles and microspheres in simulated pylorus.

Movie S3. The performance of microbubbles and microspheres in 3D printed stomach

Figures:

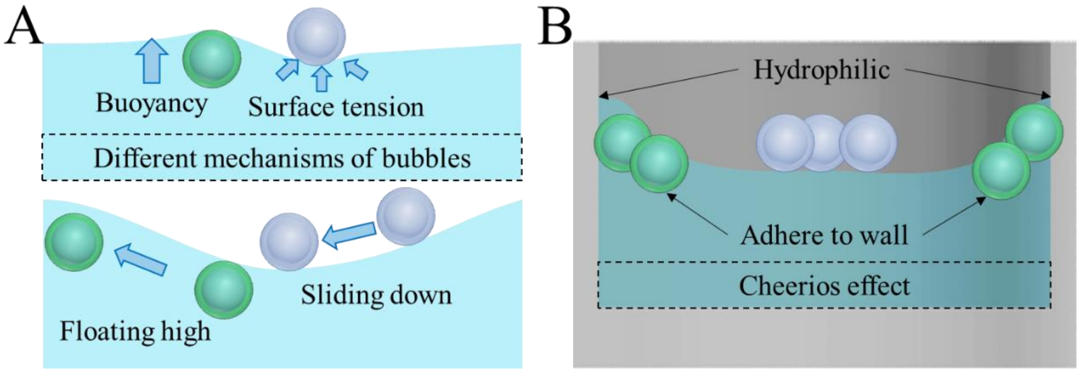

Fig. S1. **Schematic illustration of Cheerios Effect based microbubbles**. A) Cheerios Effect based microbubbles included two kinds of microbubbles. Hydrophilic microbubbles float by buoyancy and therefore tend to float to the highest part of the liquid surface, while hydrophobic microbubbles float by surface tension and therefore tend to slip to the lowest part of the liquid surface. B) In actual situations, the stomach wall is super hydrophilic, so the edge of the liquid surface in contact with the stomach wall is higher than the middle of the liquid surface, so the hydrophilic microbubbles tend to actively adhere to the stomach wall.

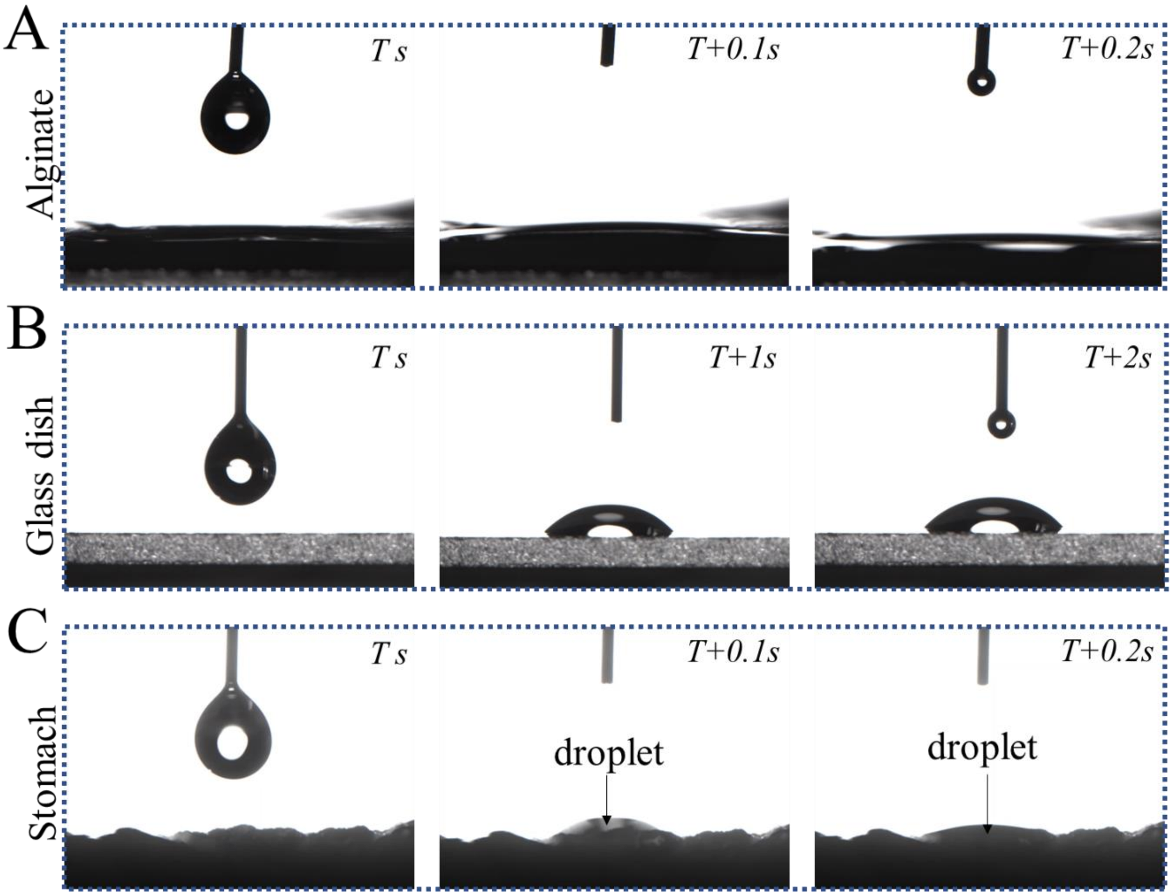

Fig. S2. **The water contact angle of alginate, stomach tissue and glass dish.** A) The water contact angle result of alginate in the hydrophilicity test. B) The contact angle change of the stomach in the hydrophilicity test; C) The water contact angle result of the glass dish.

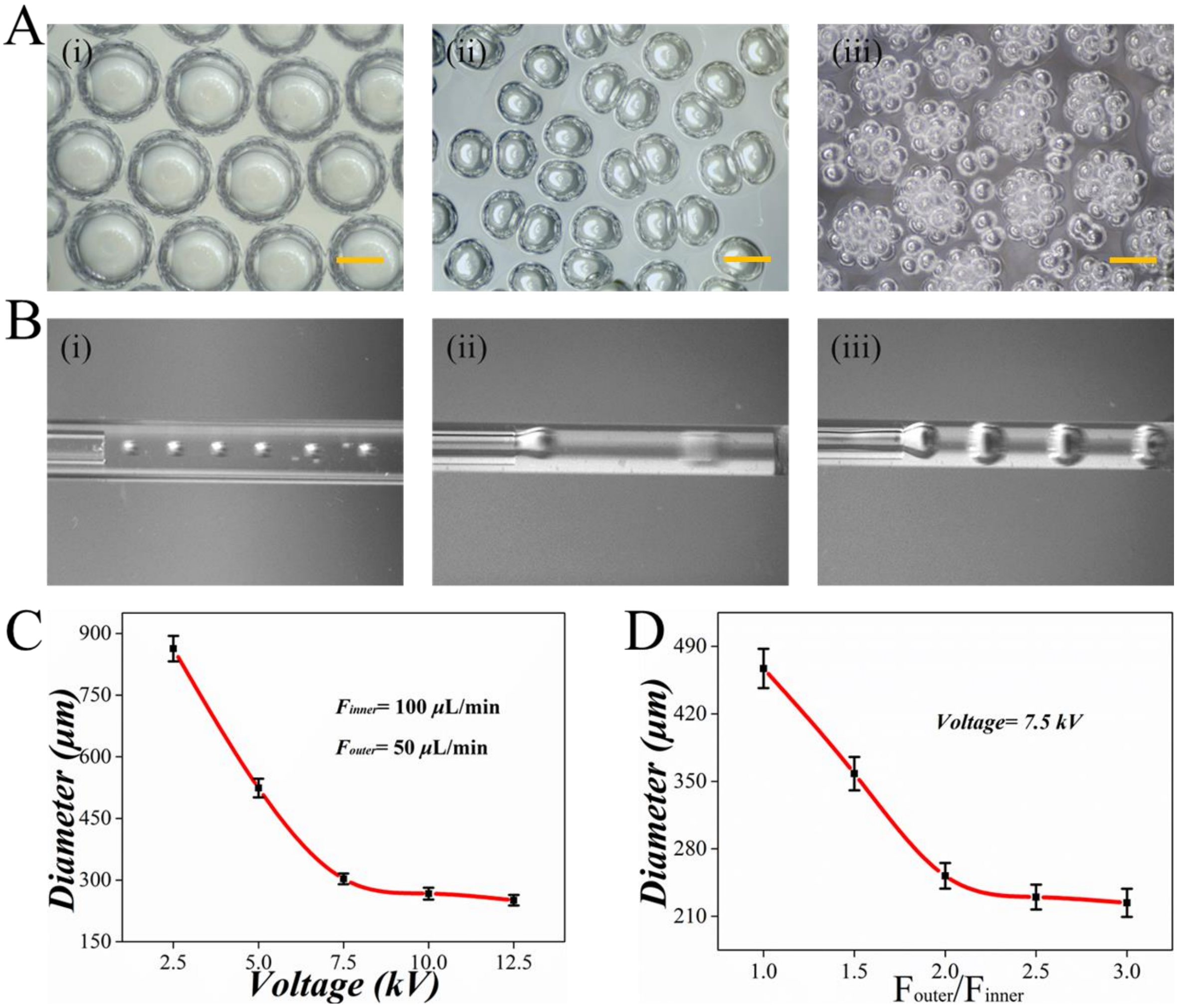

Fig. S3. **The relationship between the microbubble's morphology and producing parameters.** A) Different morphology of microbubble carriers produced at different voltages including (i) 7.5 kV, the scale bar is 200 μm; (ii) 5 kV, the scale bar is 300 μm; (iii) 2.5 kV, the scale bar is 400 μm. B) The size of microbubbles could be adjusted by the ratio of the outer phase flow rate (F_{outer}) to the inner phase flow rate (F_{inner}). C) The relationship between the diameter of microbubble carriers and voltage. D) The relationship between the diameter of the microbubble and F_{outer}/F_{inner}.(n=20).

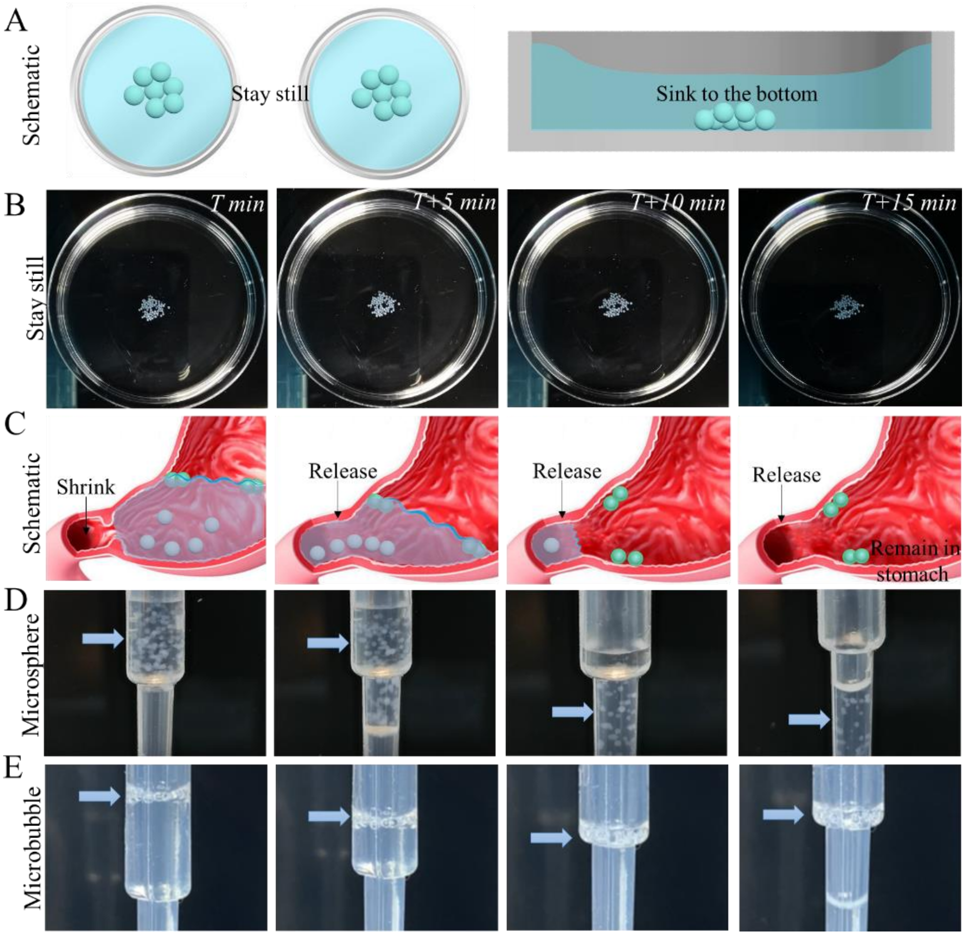

Fig. S4. **The performance of the solid microspheres in the glass dish and the simulated pylorus.** A) The schematic illustration of microspheres in liquid. B) Real-time images of the microsphere in the dish. C) Schematic illustration of microcarriers passing through pylorus. D) Analysing the behaviour of microspheres in the simulated pylorus. D) Analysing the behaviour of microbubbles in the simulated pylorus.

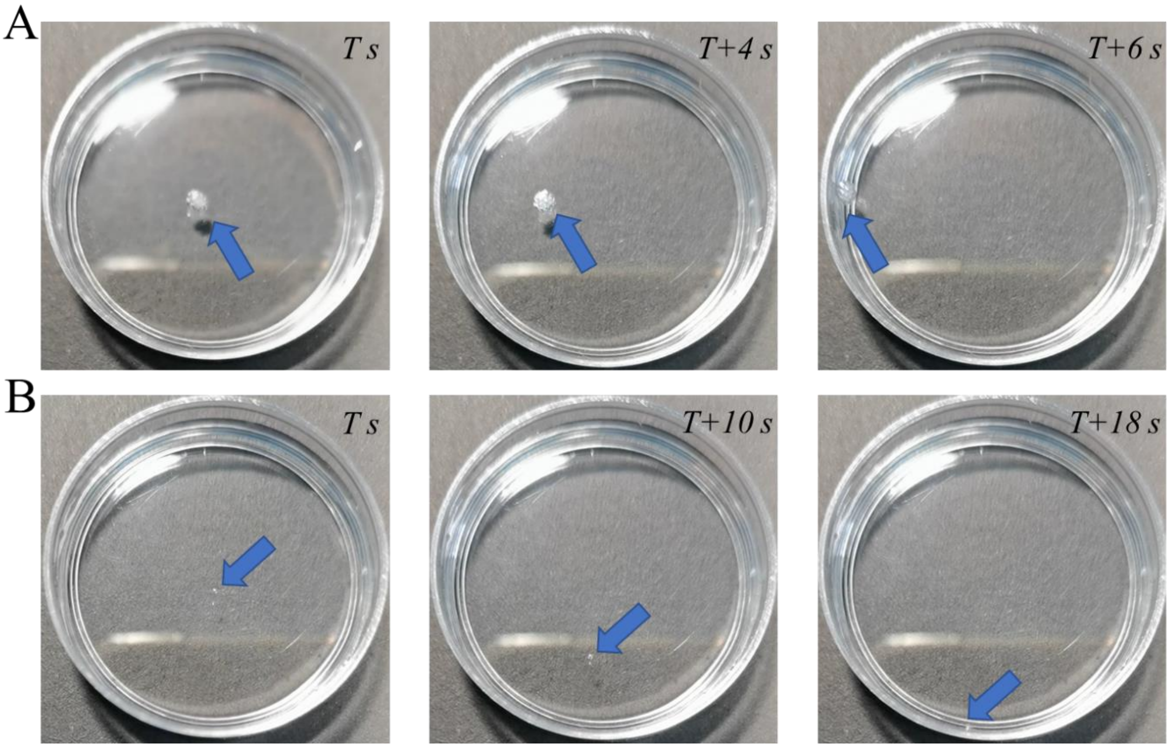

Fig. S5. The performance of microbubbles with different sizes in the dish. A) Real-time images of the larger microbubble in the dish. B) Real-time images of the smaller microbubble in the dish.

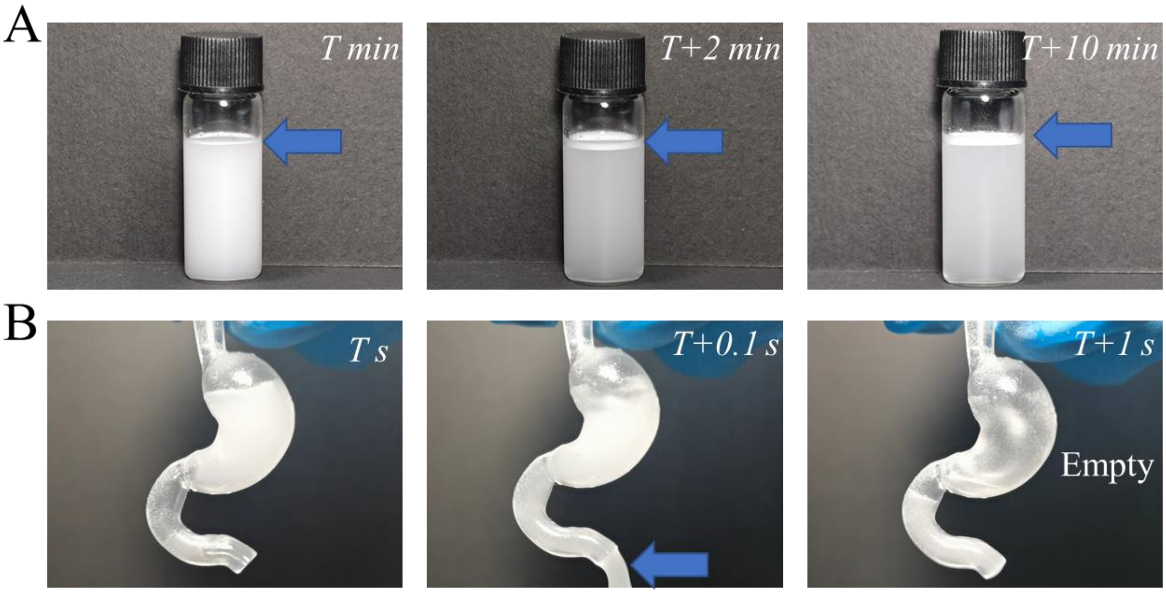

Fig. S6. The flow dynamics and stomach adhesion ability of SonoVue. A) The flow dynamics of SonoVue was tested in 10 mins. B) The adhesive ability of SonoVue in the stomach.

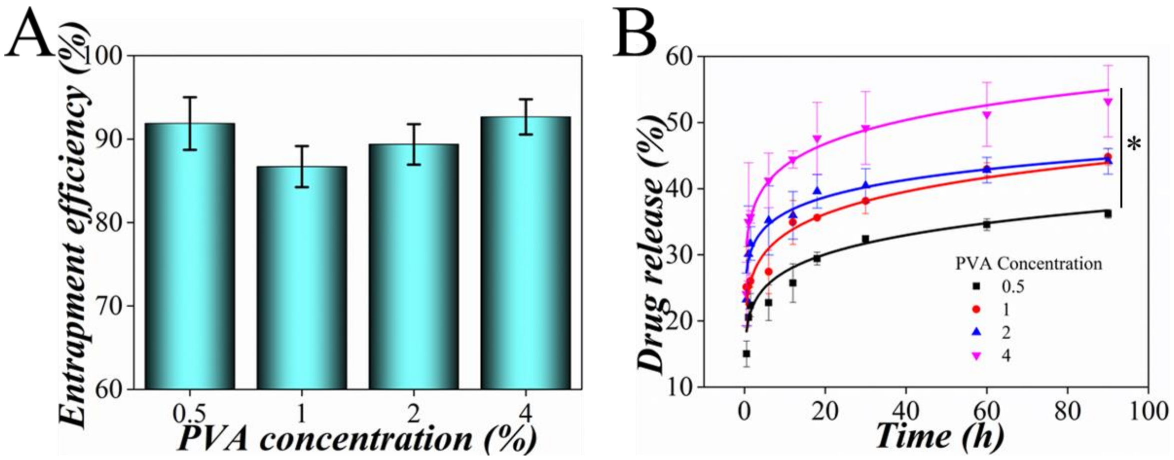

Fig. S7. The influence of PVA on entrapment efficiency and drug release. A) Entrapment efficiency of microbubbles with different concentrations of PVA. B) The drug release abilities of microbubbles with different concentrations of PVA. (T-test was used for measuring the difference between two groups, n=3, *P<0.05)

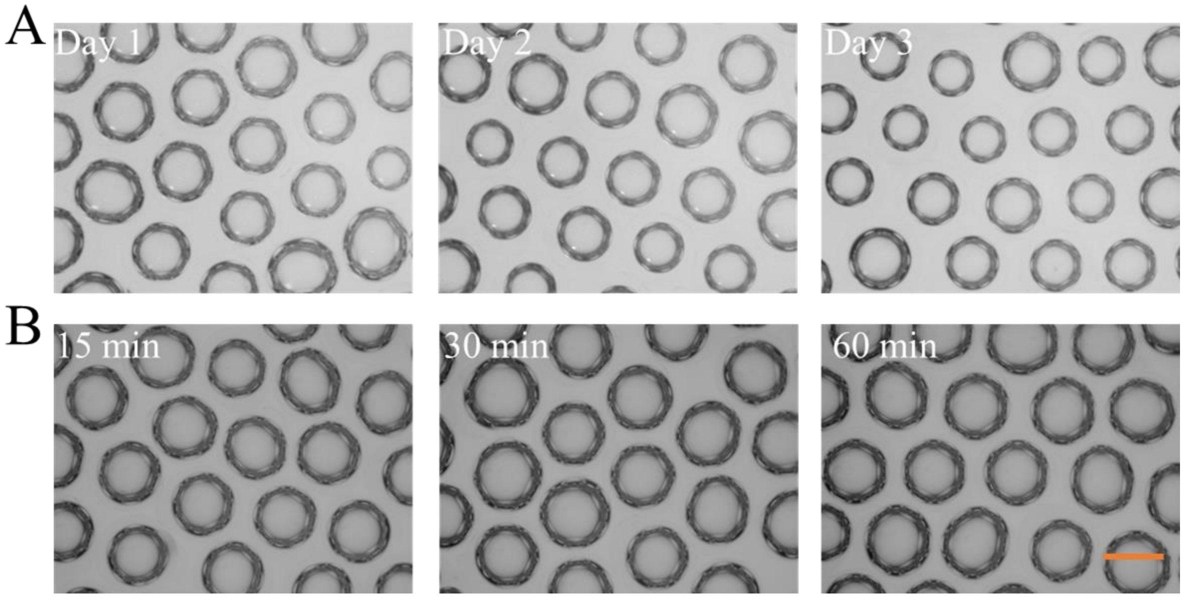

Fig. S8. The morphology of microbubbles after culturing in static state for 3 days and in digestion for 60 mins. A) The microbubbles were cultured in SGF for 3 days and images were taken on day 1, day 2 and day 3. B) The microbubbles were cultured in simulated

digesting SGF and images were taken at 15, 30 and 60 mins. The scale bar is 300 μm.

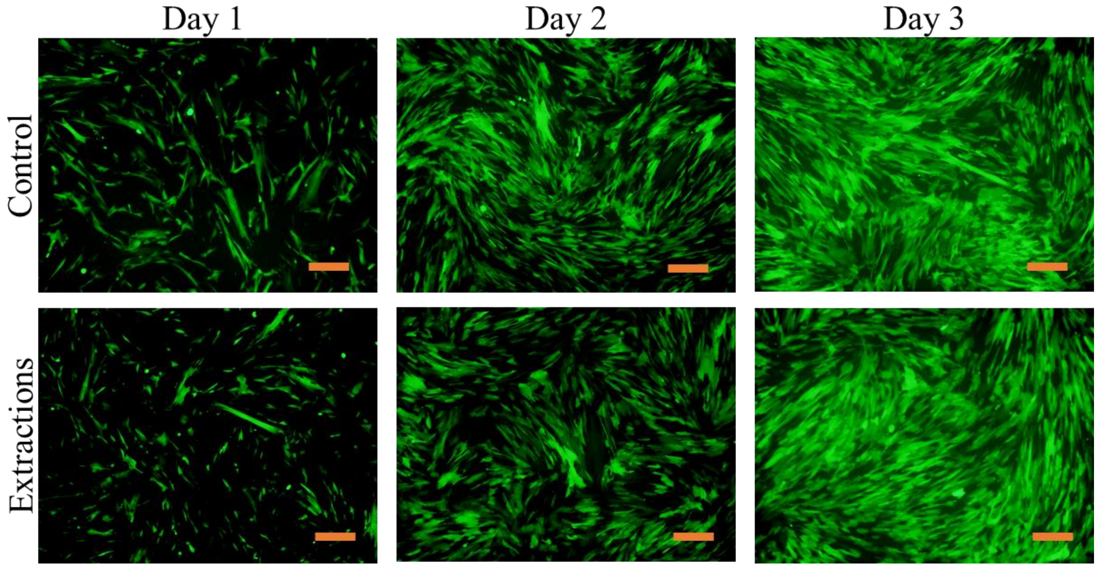

Fig. S9. **Biocompatibility of encapsulating materials.** Extractions were cocultured with cells for 3 days. The scale bars are 100 μm.

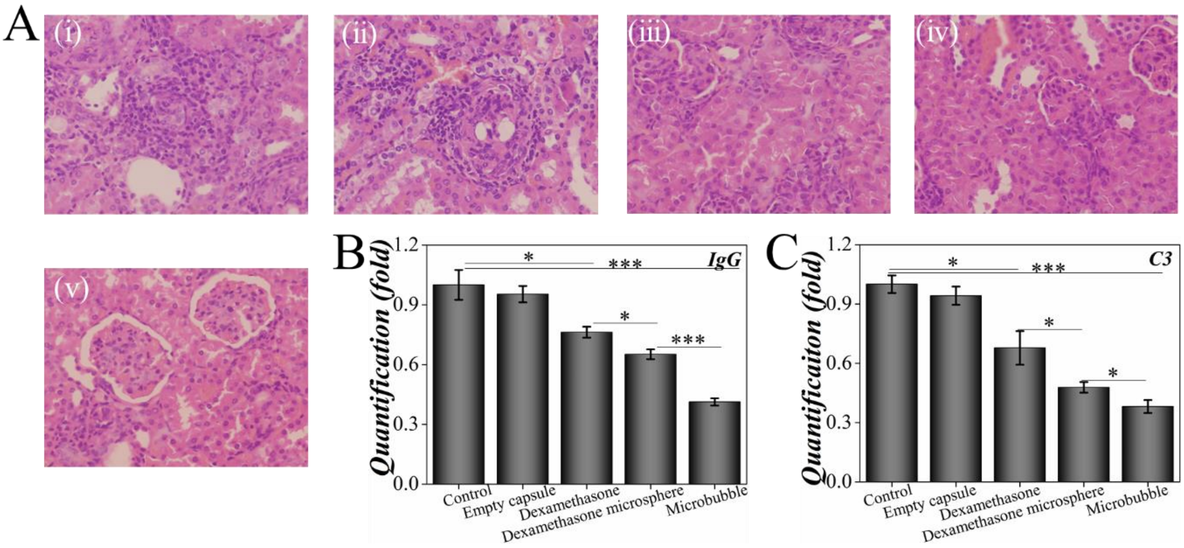

Fig. S10. Effect of microbubbles on MRL/*lpr* mice. A) Representative images of kidney HE staining after treatment. (i) Control group. (ii) Empty capsule group. (iii) Dexamethasone group. (iv) Dexamethasone group. (v) Microbubble group. B) Fluorescence quantification of IgG. C) Fluorescence quantification of C3 (T-test was used for measuring the difference between two groups, n=3, *P<0.05, ***P<0.001).
